# tDCS over the left prefrontal Cortex improves mental flexibility and inhibition in geriatric inpatients with symptoms of depression or anxiety: A pilot randomized controlled trial

**DOI:** 10.3389/fresc.2022.997531

**Published:** 2022-10-25

**Authors:** Mathieu Figeys, Sheryn Villarey, Ada W. S. Leung, Jim Raso, Steven Buchan, Hubert Kammerer, David Rawani, Megan Kohls-Wiebe, Esther S. Kim

**Affiliations:** ^1^ Faculty of Rehabilitation Medicine, University of Alberta, Edmonton, AB, Canada; ^2^ Alberta Health Services, Edmonton, AB, Canada; ^3^ Neuroscience and Mental Health Institute, University of Alberta, Edmonton, AB, Canada; ^4^ Department of Occupational Therapy, University of Alberta, Edmonton, AB, Canada; ^5^ Faculty of Kinesiology, Sport, and Recreation, University of Alberta, Edmonton, AB, Canada; ^6^ Department of Psychiatry, Faculty of Medicine and Dentistry, University of Alberta, Edmonton, AB, Canada; ^7^ Department of Communication Sciences and Disorders, University of Alberta, Edmonton, AB, Canada

**Keywords:** transcranial direct current simulation, executive functioning, depression, anxiety, geriatric, rehabilitation, mental health, neuromodualtion

## Abstract

**Background:**

Patients with depression and/or anxiety are commonly seen in inpatient geriatric settings. Both disorders are associated with an increased risk of cognitive impairments, notably in executive functioning. Transcranial direct current stimulation (tDCS), a type of non-invasive brain stimulation, involves the administration of a low-dose electrical current to induce neuromodulation, which ultimately may act on downstream cognitive processing.

**Objective:**

The purpose of this study was to determine the effects of tDCS on executive functioning in geriatric inpatients with symptoms of depression and/or anxiety.

**Design:**

Pilot Randomized Controlled Trial.

**Setting:**

Specialized geriatric wards in a tertiary rehabilitation hospital.

**Methods:**

Thirty older-aged adults were recruited, of which twenty completed ten-to-fifteen sessions of 1.5 mA anodal or sham tDCS over the left dorsolateral prefrontal cortex. Cognitive assessments were administered at baseline and following the tDCS protocol; analyses examined the effects of tDCS on cognitive performance between groups (anodal or sham tDCS).

**Results:**

tDCS was found to increase inhibitory processing and cognitive flexibility in the anodal tDCS group, with significant changes on the Stroop test and Trail Making Test-Part B. No significant changes were observed on measures of attention or working memory.

**Discussion:**

These results provide preliminary evidence that tDCS-induced neuromodulation may selectively improve cognitive processing in older adults with symptoms of depression and/or anxiety.

**Clinical Trials Registration:**

www.clinicaltrials.gov, NCT04558177

## Introduction

Older adults admitted for rehabilitation often present with concomitant depression and/or anxiety (1–3). Multi-morbidity as well as cognitive impairment may be synergistically coupled with depression and anxiety in older adults (4, 5). Additionally, depression and anxiety are often comorbid psychiatric disorders (6, 7). In older adults, the severity of depression often increases when an anxiety disorder comorbidity is present (8, 9). Furthermore, depression and/or anxiety may impair processes related to successful rehabilitation and are associated with increased length of hospital stay, increased utilization of inpatient resources, and a higher rate of inpatient mortality (2, 10, 11).

Executive functioning (EF) is a key mediating factor associated with functional status in older adults (12). EF is often discussed in terms of three subdomains required to perform daily activities: inhibition, working memory, and cognitive flexibility (13). Although EF naturally declines across normal cognitive ageing (14–16), numerous underlying etiologies including mild cognitive impairment (MCI) and dementia can impair EF beyond the extent seen across normal cognitive ageing (17). Cognitive changes in EF have been observed in older adults with neurocognitive and psychiatric disorders, including depression, anxiety, MCI, and dementia (18–26). Therefore, interventions aimed at improving EF may lead to improved functional outcomes in older adults [See Karr and colleagues (27) for a review].

Experimental use of transcranial direct current stimulation (tDCS) has been increasingly explored as a cognitive enhancement technique, including within older adult populations (28–30). tDCS is an emerging method of non-invasive brain stimulation, where neuromodulation is achieved by altering neuronal polarities through the administration of a low-dose electrical current applied across the scalp to target brain structures. Although transcranial magnetic stimulation (TMS) has been approved for clinical applications such as treatment-resistant depression in an increasing number of countries, tDCS approval currently remains primarily for research purposes in most nations (31).

Previous studies have demonstrated that tDCS can result in improvements in cognition, as well as modulating symptoms associated with depression, MCI, and dementia (32–34). Several of these studies explored the effects of tDCS on the dorsolateral prefrontal cortex (DLPFC) (32, 34). The DLPFC has also been established as a neural region involved in mediating cognitive processes underlying EF, including attention (35), cognitive flexibility [the ability to switch across multiple different concepts in a context-dependent manner; (36, 37)] and higher-order cognition (38, 39). Further, neuroimaging has consistently demonstrated that the DLPFC is implicated in depression and anxiety (40–45).

The purpose of this pilot study was to explore the effects of tDCS on cognitive performance in older adult inpatients with symptoms of depression and/or anxiety within an inpatient geriatric rehabilitation clinical care setting. The following research question was of interest: *What are the effects of multi-session anodal tDCS over the left DLPFC on EF in older adult inpatients with symptoms of depression and/or anxiety compared to those receiving sham stimulation?* It was hypothesized that anodal tDCS would augment performance across executive functioning processes compared to sham stimulation.

## Materials and methods

To answer the research question, a double-blinded parallel, sham-controlled, single-centre randomized control trial was conducted at the Glenrose Rehabilitation Hospital (Alberta Health Services, Edmonton, Canada). Ethics was approved by the University of Alberta Research Ethics Board (Pro00078317). The study protocol was registered with the National Institute of Health (NCT04558177).

### Participants

A subset of patients taking part in a larger study examining the effects of tDCS in geriatric depression and anxiety were recruited to undergo additional cognitive assessments before and after tDCS stimulation. Convenience sampling was utilized; participants were recruited from Specialized Geriatric Rehabilitation inpatient wards at the Glenrose Rehabilitation Hospital (Alberta Health Services, Edmonton, Canada). All patients underwent depression and anxiety screenings upon admission using the Geriatric Depression Scale [GDS; (46)] and Geriatric Anxiety Inventory [GAI; (47)] These screening tests were administered by Occupational Therapists on the geriatric wards. Inclusion criteria were defined as: being over the age of 65 years old, GDS ≥ 5, GAI ≥ 8, proficiency in English, ability to provide informed consent, and the absence of dementia. If eligible for participation, patients were referred to the research team by the Occupational Therapists or Physicians for recruitment. Signed informed consent was obtained from patients who agreed to participate in the study or their respective powers of attorney. Exclusion criteria included: active infection, implanted medical devices (e.g., cardiac pacemakers, deep brain stimulators), history of seizures, metallic implants in the head, or history of severe neurological illness. Participation in the study was in addition to usual routine clinical care, and participants did not receive compensation.

### Clinical care

Multi-disciplinary clinical care varied across patients; however, all patients received a combination of geriatric-oriented physical and occupational therapy, in addition to medical and nursing care. As routine clinical care remained the primary focus for these patients, tDCS sessions were occasionally skipped if required to accommodate standard patient care. Therefore, not all individuals were able to complete all fifteen tDCS sessions; participants who completed more than 10 tDCS sessions were included for analysis.

### Cognitive assessments

Participants underwent paper-based cognitive assessments administered by the primary author, with a battery of tasks largely assessing EF, including: inhibitory control, working memory, attention, processing speed, and cognitive flexibility (see Table 1 for an overview of the cognitive battery administered). The cognitive battery was administered in the same order across participants. Instructions and practice trials of the assessments were given to ensure participant comprehension; errors were immediately corrected during the trial runs. To minimize potential physiological and circadian confounds relating to cognitive fatigue, cognitive assessments and tDCS sessions were administered between 15:00–17:30 daily based on the participant's availability around their clinical care routine. Cognitive testing and tDCS sessions were delivered in participants' hospital rooms with distractions minimized (e.g., lights on, television off, door closed); attention and comprehension of the cognitive tasks were ensured by completing practice trials.

**Table 1:** Overview of the cognitive battery administered.

Cognitive assessment	Domains targeted	Task description	Scoring
SDMT (48)	Processing Speed, attention	Participants matched a series of symbols to a numbered answer key	Number of correct responses divided by the number of total responses in a 90 s period
TMT-A (49)	Processing speed, visuospatial attention	Participants connected a series of circled numbers ranging from 1 to 25 scattered randomly across the page by drawing a line in sequential order	Total amount of time (seconds)
TMT-B (49)	Cognitive flexibility, task switching processing speed, visuospatial attention	Starting at 1, participants connected a series of circled numbers to the corresponding circled letter by drawing a line in sequential order. (e.g.: 1-A-2-B-3-C…)	
Digit Span-Forwards (50)	Short-term memory	Participants were verbally presented with a sequence of numbers and asked to repeat the sequence, with each successive attempt requiring a longer sequence of numbers (Two attempts per sequence)	If a participant was correct the first time, one point is awarded; if a participant was wrong on the first attempt but was correct on the second attempt, a score of zero was awarded. The test was discontinued once two wrong attempts on the same sequence occurred.
Digit Span-Backwards (50)	Working Memory	Participants were verbally presented with a sequence of numbers, and asked to repeat the sequence in reverse order, with each successive attempt requiring a longer sequence of numbers (Two attempts per sequence)	
Stroop Task-Interference Scores (51)	Executive function-inhibitory control	A standardized Stroop test was administered. It is thought that interference scores provide information about processing speed and inhibitory control (51)	The participant was given 45 s to complete each Stroop subtest. Interference scores were calculated, given by: $I=CW-\frac{\left(W\;\times \;C\right)}{\left(W\;+\;C\right)}$

SDMT, Symbol Digit Modalities Test; TMT-A, Trail Making Test (Part A); TMT-B, Trail Making Test (Part B); I, Interference; C, Colour; W, Word; CW, Colour-Word.

### tDCS randomization & parameters

To maintain double-blinding, six HDCStim tDCS devices (Newronika, Italy) were programmed to deliver anodal (*n* = 3) or sham (*n* = 3) stimulation by an individual not involved in the study. Participants, researchers, and clinicians remained blinded to the intervention. Recruited participants were allocated a specific tDCS device for the study in a 1:1 allocation ratio. Simple randomization was performed, dependent on the programming of the tDCS device to deliver an active anodal or sham stimulation.

**Figure 1:**
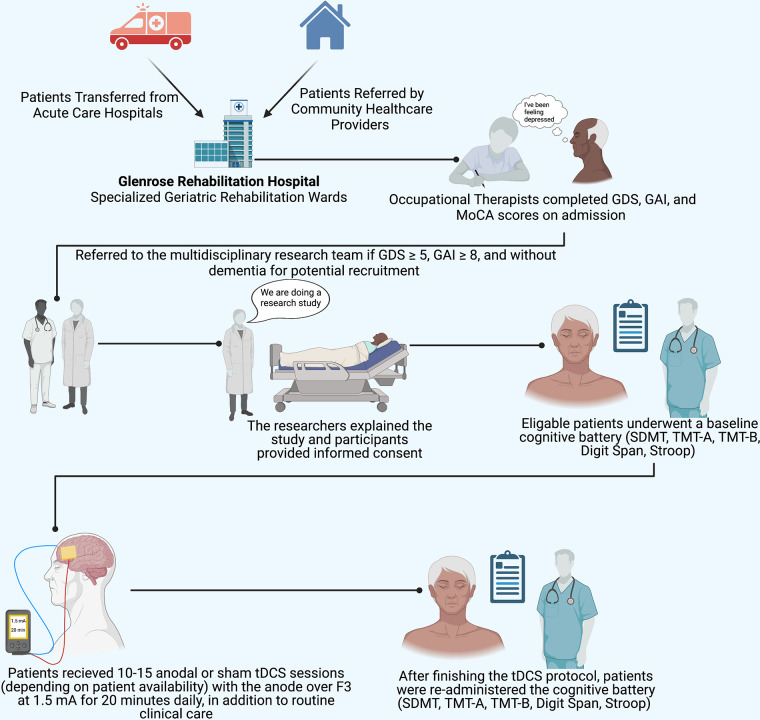
Implemented protocol. The study protocol implemented illustrating participant recruitment, consent, assessment, and tDCS procedures. SDMT, symbol digit modalities test; TMT-A, Trail Making Test Part A; TMT-B, Trail Making Test Part B; MoCA, Montreal Cognitive Assessment. tDCS electrode placement: the anode (red) was placed over F3, the cathode (blue) was placed over the right supraorbital region. Figure 1 was created with Biorender.com.

tDCS sessions were delivered daily, based on the participants' availability around routine clinical care, with participants receiving 10–15 consecutive sessions (including weekends). tDCS parameters were based on previously established safety parameters [refer to (52)]. tDCS sessions were administered by the primary author as well as three other research assistants, who all received training from the same rehabilitation engineer familiar with tDCS. Electrodes were placed in 5 cm × 7 cm (35 cm^2^) electrode sponges and saturated with 10 ml 0.9% NaCl solution and secured to the scalp using a snuggly fitting hairnet. Using the 10:20 EEG system (53), the anode was placed over F3 (the left DLPFC) and the cathode over the contralateral (right) supraorbital region, in line with Liao and colleagues (54); refer to Figure 1. A 1.5 mA current was applied for 20 min per session. The current was ramped over 1 min until reaching 1.5 mA (52). Participants were free to participate in any task during tDCS sessions; most participants remained in bed or watched television.

The sham group received 1.5 mA of electrical stimulation for one minute: ramping up over 15 s, steady for 30 s and ramping down for 15 s. This blinding technique involves the replication of a cutaneous electrical sensation used to mask participants' group allocation (52, 55). Once the data collection period was completed, stimulator assignment and group allocation (anodal, sham) were revealed by the individual outside of the study.

### Analyses

The primary outcome was examining the effects of tDCS on cognitive performance in older adults with symptoms of depression or anxiety. Differences between pre-test and post-test scores were calculated for all cognitive assessments. Analysis methods were based on a previous randomized control trial using multi-session tDCS using a similar sample size of older adults with MCI (54). A two-way mixed ANOVA was performed in SPSS (Version 27, IBM Corp. Armonk, NY) on change scores, with time as a within-subjects factor and treatment condition (anodal or sham) as a between-subject factor. An alpha of 0.05 was set to determine significance, with a Bonferroni correction for multiple comparisons. Significant main effects and main interactions reported in the two-way mixed ANOVA were followed up using both paired and independent *t*-tests to determine any significant differences. Analysis was conducted based on the tDCS group assignment.

## Results

### Participants

Twenty eligible participants were included in the final sample (*n* = 10 in each group), consistent with similar sample sizes of older adults with multi-session tDCS interventions in MCI and depression (54, 56). Participant recruitment and data collection were stopped early because of the emergence of SARS-CoV-2. The recruitment process is highlighted in Supplementary Figure S1. Admitting diagnoses included: decreased functional mobility and weakness, falls, cognitive decline, depression, anxiety, cancer, congestive heart failure, chronic obstructive pulmonary disease, myocardial infarction, and cerebrovascular accidents.

**Figure 2:**
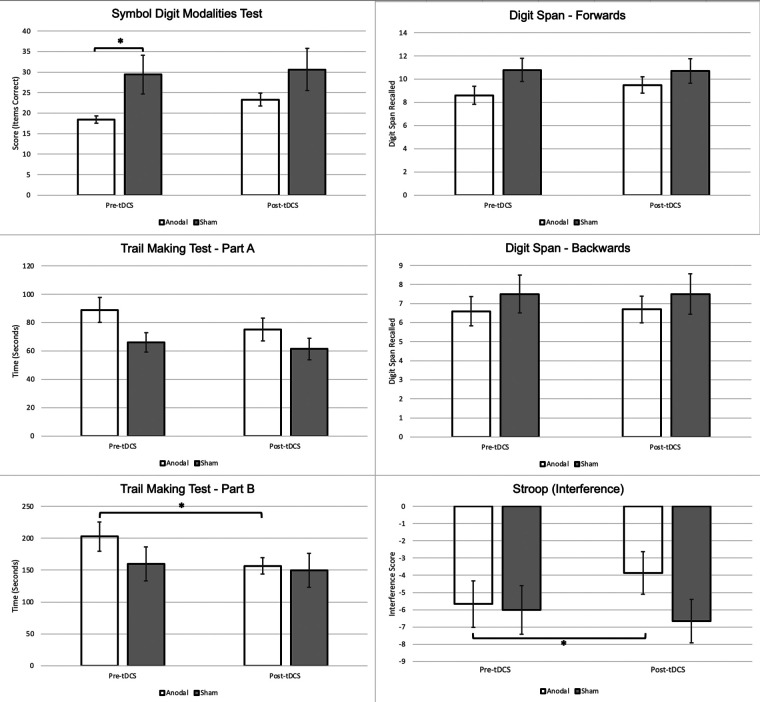
Changes in cognitive performance between anodal & sham stimulation groups. Note: * is significant at *p* < 0.05. Stroop Interference: Negative interference suggests a pathological impairment of inhibition, lower scores indicate greater impairment (60).

No significant differences were found between those who received 10–14 tDCS sessions and those who completed all 15 sessions, as well as on age, education, baseline MoCA, GDS, and GAI scores. Hence, we combined the results of those receiving 10–15 tDCS sessions and report them together, respective to their tDCS group allocation. An independent *t*-test found no significant differences between the active and sham groups on the variables of age, years of education, baseline MoCA, GDS, and GAI (all *p's* > 0.05; Refer to Table 2). An overview of obtained results is presented in Figure 2.

**Table 2:** Participant demographics & cognitive battery performance.

Assessment	*Pre-tDCS* (Mean ± SD)			*Post-tDCS* (Mean ± SD)			* *	* *
	Anodal	Sham	*p*-value	Anodal	Sham	*p-*value		
***Participant Demographics***								
Age (Years)	77.10 ± 6.98	72.50 ± 7.46	0.172					
Education (Years)	11.60 ± 2.01	12.70 ± 3.97	0.445					
Biological Sex (Males/Females)	6/4	4/6						
*Baseline Screenings*							***Psychometrics***	
Baseline GDS	7.70 ± 2.98	9.20 ± 3.52	0.404				Normal: 0–4 Mild: 5–9 Moderate-to-Severe: 10+	
Baseline GAI	11.70 ± 5.19	8.40 ± 6.43	0.158				Normal: 0–7 Query Anxiety: 8+	
Baseline MoCA	22.20 ± 3.36	23.30 ± 4.06	0.518				Normal: 26+ Cognitive Impairment Mild: 18–25 Moderate: 10–17 Severe: <10	
Baseline OPQoL	115.33 ± 16.8	113.30 ± 11.48	0.403				Range: 35–175 *Lower scores indicative of lower quality of life	
***Cognitive Battery***							***Normative Data (Mean ± SD)***	
SDMT (Items Correct)	***18.40 *** **±** *** 2.84***	29.40 ± 14.95*	**0.035**	23.30 ± 5.01	30.60 ± 16.24	0.191	Anodal	29.76 ± 10.65
							Sham	34.79 ± 10.54
Trail Making Test Part A (Seconds)	88.90 ± 27.79	66.00 ± 25.69	0.072	75.20 ± 21.76	61.40 ± 24.15	0.196	Anodal	50.81 ± 17.44
							Sham	40.13 ± 14.48
Trail Making Test Part B (Seconds)	202.70 ± 72.68	159.70 ± 83.88	0.236	156.60 ± 40.06	149.70 ± 83.59	0.410	Anodal	130.61 ± 45.74
							Sham	86.27 ± 24.07
Digit Span-Forward (Span)	8.60 ± 2.76	10.80 ± 3.16	0.114	9.50 ± 2.22	10.70 ± 3.34	0.178	Anodal	4.98 ±* 0.97*
							Sham	5.39 ±* *1.07
Digit Span-Backward (Span)	6.60 ± 2.46	7.50 ± 2.95	0.468	6.70 ± 2.21	7.50 ± 2.67	0.238	Anodal	3.46 ±* 0.99*
							Sham	3.80 ±* 1.08*
Stroop (Interference)	−5.67 ± 4.24	−6.01 ± 4.49	0.861	−3.86 ± 3.90	−6.65 ± 3.98	0.131	Anodal	*
							Sham	

*p*-values obtained from independent *t*-tests. MoCA, Montreal Cognitive Assessment; GDS, Geriatric Depression Scale; GAI, Geriatric Anxiety Inventory; SD, Standard Deviation. Normative data was obtained from: SDMT (57); Trail Making Test Parts A & B (58); Digit Span (59); Stroop*: Negative interference suggests a pathological impairment of inhibition, lower scores indicate greater impairment (60). Age and education adjusted normative data is reported. Psychometric data taken from: GDS (61); GAI (47); MoCA (62); OPQoL (63).

### Symbol digit modalities test (SDMT)

A non-significant main effect of time [*F* (1,18) = 3.031, *p* = 0.099, *ηp*^2^ = 0.144] and condition [*F* (1,18) = 0.53, *p* = 0.475, *ηp*^2^ = 0.029] was found on the SDMT, as well as a non-significant time × condition (anodal or sham) interaction [*F* (1,18) = 2.82, *p* = 0.110, *ηp*^2^ = 0.136]. Paired *t*-tests demonstrate a significantly higher post-tDCS SDMT score when compared to the baseline scores within the anodal group only, however, no significant group differences were found on the independent *t*-test.

### Trail making test part A (TMT-A)

Although there was no main effect of condition on TMT-A [*F* (1,18) = 2.83, *p* = 0.110, *ηp*^2^ = 0.136], there was a significant effect of time [*F* (1,18) = 15.96, *p* < 0.001, *ηp*^2^ = 0.470]. The time × condition interaction for TMT-A was found to be non-significant [*F* (1,18) = 3.95, *p* = 0.062, *ηp*^2^ = 0.180]. Paired *t*-tests demonstrated significant improvement in TMT-A times within both groups, and an independent *t*-test found that the change from baseline to post-tDCS was significantly greater in the anodal group relative to the sham group.

### Trail making test part B (TMT-B)

No significant main effect of condition on TMT-B completion time was found [*F* (1,18) = 0.64, *p* = 0.435, *ηp*^2^ = 0.034]. The main effect of time on TMT-B completion time was significant [*F* (1,18) = 10.70, *p* = 0.004, *ηp*^2^ = 0.373]. The time × condition interaction was determined to be significant [*F* (1,18) = 4.44, *p* = 0.049, *ηp*^2^ = 0.198]. The paired *t*-test found that only the anodal group demonstrated a significant difference in TMT-B time between baseline and post-tDCS. The independent *t*-test demonstrated a significant improvement in TMT-B scores within the anodal group.

### Digit span forward

No significant main effects of time [*F* (1,18) = 2.23, *p* = 0.152, *ηp*^2^ = 0.110] and condition [*F* (1,18) = 1.80*, p* = 0.197, *ηp*^2^ = 0.091] were observed. Interactions between time and condition on digit forward scores [*F* (1,18) = 3.49, *p* = 0.078, *ηp*^2^ = 0.162] were also non-significant.

### Digit span backwards

No significant main effect of time [*F* (1,18) = 0.11, *p* = 0.747, *ηp*^2^ = 0.006] or main effect of condition [*F* (1,18) = 0.64, *p* = 0.435, *ηp*^2^ = 0.034] was found. Like digit span forwards, there was no significant interaction between time and condition [*F* (1,18) = 0.11, *p* = 0.747, *ηp*^2^ = 0.006]. In addition, no significant within-group or between-group differences were noted across *t*-tests.

### Stroop interference

A non-significant main effect of time [*F* (1,18) = 2.93, *p* = 0.104, *ηp*^2^ = 0.140] and a non-significant main effect of condition [*F* (1,18) = 3.95, *p* = 0.062, *ηp*^2^ = 0.180] was found. A significant time × condition interaction was found on the two-way ANOVA [*F* (1,18) = 4.77 *ηp*^2^, *p* = 0.042, *ηp*^2^ = 0.209]; significant increases in Stroop interference scores were found in the paired *t*-test only within the anodal group. In addition, there was a significant difference between the changes in interference score from pre-tDCS to post-tDCS between the active and sham groups, with the active group demonstrating a greater mean change.

## Discussion

In this study, 20 older adult inpatients with self-reported symptoms of depression and/or anxiety received 10–15 anodal or sham tDCS sessions delivered over the left DLPFC. We report that the tDCS-protocol provided over the left DLPFC selectively augmented cognitive processing. In line with our hypothesis, we note significant changes in tests involved in higher cognitive processes (i.e., the TMT-B and Stroop tasks). However, the effectiveness of tDCS on the domains of attention and working memory was minimal.

The time required to complete the TMT-B decreased in the anodal group, suggestive of increased cognitive flexibility and interference processing. It is generally agreed that the TMT-B has a higher sensitivity to central EF and cognitive flexibility and task-switching compared to the TMT-A (64, 65). Increased performance on the Stroop task was also evident, suggesting a potential increase in inhibition capacity. tDCS may have modulated the ability to minimize interfering distractors, resulting in more accurate and rapid processing of presented stimuli. In addition, we report null tDCS effects on the other DLPFC-associated cognitive processes of working memory and attention, assessed by the SDMT, TMT-A, and the digit span tests. Cognitive flexibility, working memory, and inhibition are processes highly involved in EF (13), and necessary in higher-order cognitive processing. Thus, tDCS-induced neuromodulation may have invoked a selective-synergistic interaction in EF domains of cognitive flexibility and interference, increasing performance on tests of higher-order cognition.

These results corroborate previously reported findings. In a similar study design targeting Parkinson's Disease, Doruk and colleagues (66) report significant improvements on the TMT-B without changes on the TMT-A, with the maintenance of these findings extending to one-month post-stimulation. Bystad and colleagues (67) further report significant gains on the TMT-B without improvements in the TMT-A only within a young adult group. In addition, Loftus and colleagues (68) report reaction time improvements on the Stroop task in young adults after receiving anodal DLPFC stimulation resulting in inhibitory control enhancement; our results continue to support inhibition control enhancement in older aged adults. However, the extent of tDCS effects on cognitive enhancement may vary across the lifespan, which to date remains largely unknown.

We report null tDCS-induced cognitive effects on working memory and attention. These findings are in line with Kumar and colleagues (69) who report a lack of tDCS effects on working memory and global cognition in older adults with depression. Nonetheless, contrasting results are reported by Nissim and colleagues (70) who reported significant changes in working memory as well as functional connectivity after a two-week tDCS protocol paired with cognitive training in healthy older adults. Again, the pairing of tDCS with working memory training has also been demonstrated to increase digit span performance in older adults (71). Although tDCS alone has been demonstrated to selectively modulate working memory within older adults with higher levels of education (72), dual tDCS-cognitive training paradigms may optimize effects on working memory and attention which requires further investigation.

Taken together, these results further contribute to the proposed roles of the DLPFC in higher-order cognition and behaviour (73, 74). However, the role of the DLPFC in lower-order cognition remains unclear. Other frontal lobe structures, including the ventrolateral prefrontal cortex, have been proposed to be engaged in lower-order cognitive processing (75). In addition, these results provide additional evidence that tDCS may selectively increase EF in older adults. Future research is needed to determine who may be optimal candidates for tDCS therapy for EF augmentation, and the effects of tDCS on prefrontal networks.

### Limitations and future directions

Across the literature, varying study designs, cognitive protocols, populations of interest, and tDCS parameters exist. The effects of tDCS may be task-specific, cognitive-domain specific, age, and etiology dependent, with varying montages resulting in varying neuro-cognitive modulatory effects. Furthermore, additional factors including multi-morbidity, level of education, and pharmacological agents may all impact neurological and cognitive modulation, which was not accounted for in this study. Taken together, the generalizability of the obtained results to other clinical populations, age groups, tDCS montages, and cognitive domains should be interpreted with caution.

The tDCS montage applied in this study (anode over the left DLPFC; cathode over the contralateral supraorbital region) was similar to a separate randomized control trial using tDCS in older adults with MCI (54). However, previous tDCS modelling highlighted the potential of deeper cortical and larger white matter network activation using an extracephalic return electrode (76), which in turn may alter the efficacy of tDCS and the obtained results. In addition, morphological changes and cerebral atrophy present in MCI may impact tDCS current vectors and electrical field densities (77). Additionally, no significant differences were found between those completing 10–15 tDCS sessions. Thus, the effect of tDCS-intervention frequency and intensity on EF performance in older-adult inpatients should be investigated in future studies.

The purpose of this study was to investigate whether tDCS influenced cognition within a sample of older adult inpatients with symptoms of depression and/or anxiety. Within this study, participants had an overall mean MoCA of 22.75, which may be indicative of MCI. Furthermore, cognition may have been impaired due to the confounds related to depression and anxiety. With this study designed as a pilot, our sample may limit the overall power, however, conducting this study within the hospital context contributes towards ecological validity and generalizability of tDCS applications in real-world clinical settings with an interdisciplinary approach.

In this study, we report no major adverse effects from tDCS stimulation. tDCS was found to be well-tolerated by the older-adult participants and did not significantly interfere with routine clinical care. Mild side effects including a tingling sensation under the electrodes, as well as slight discomfort from the snuggly fitted hairnet, were reported by some individuals; these side effects are consistent with previous studies (78).

Unique considerations exist when recruiting patients from an inpatient geriatric rehabilitation setting for a tDCS study, including coordination of treatments with routine clinical care, family visits, legal factors, personal values, and comfort. These factors should be taken into consideration and weighed in terms of the feasibility of future studies. Additionally, with a pragmatic design, real-world conditions were present which may have impacted the results. For instance, during the tDCS sessions, participants were free to sleep, read, or watch television during their downtime; it is possible that the activities completed during the tDCS interventions may have impacted cognitive performance, which should be examined in future studies. Further, test-retest reliability was not assessed, which we recognize as a limitation. Additionally, future studies should incorporate larger sample sizes, consider the pairing of tDCS with cognitive or behavioural training, explore other tDCS montages, include a maintenance period, incorporate neuroimaging modalities, consider adding other executive functioning assessments, and explore effects in other cognitive disorders associated with ageing including MCI and dementia. In addition, future studies are encouraged to consider ecological validity to extend the generalizability of cognitive augmentation into real-world settings.

## Conclusion

In this study, multi-session tDCS over the left DLPFC appears to invoke beneficial cognitive augmentation within the domains of inhibition processing, processing speed, and cognitive flexibility in older adult inpatients with symptoms of depression or anxiety. This evidence supports that anodal tDCS-invoked neuromodulation may extend into cognitive modulation, including executive functioning. By examining the effects of anodal tDCS within a geriatric sample, we contribute to the ongoing investigation of non-invasive brain stimulation targeting cognitive decline in older adults. Future studies should continue to investigate tDCS in normal and pathological cognitive ageing, in addition to targeting the optimization of protocols, as well as determining ideal candidates for tDCS interventions. The results of tDCS research are important for assessing its efficacy and practicality for clinical and therapeutic use within geriatric rehabilitation settings.

## Data Availability

The raw data supporting the conclusions of this article will be made available by the authors, without undue reservation.
