# Supplementary material for: tDCS over the left prefrontal Cortex improves mental flexibility and inhibition in geriatric inpatients with symptoms of depression or anxiety: A pilot randomized controlled trial
Source: Front Rehabil Sci. 2022 Oct 25;3:997531. doi: 10.3389/fresc.2022.997531 (PMC9641275; doi:10.3389/fresc.2022.997531)
Supplement: Supplementary file 1 [file Datasheet1.docx]

***Supplementary Material***

**Supplementary Figure 1: CONSORT Flow Diagram**

**
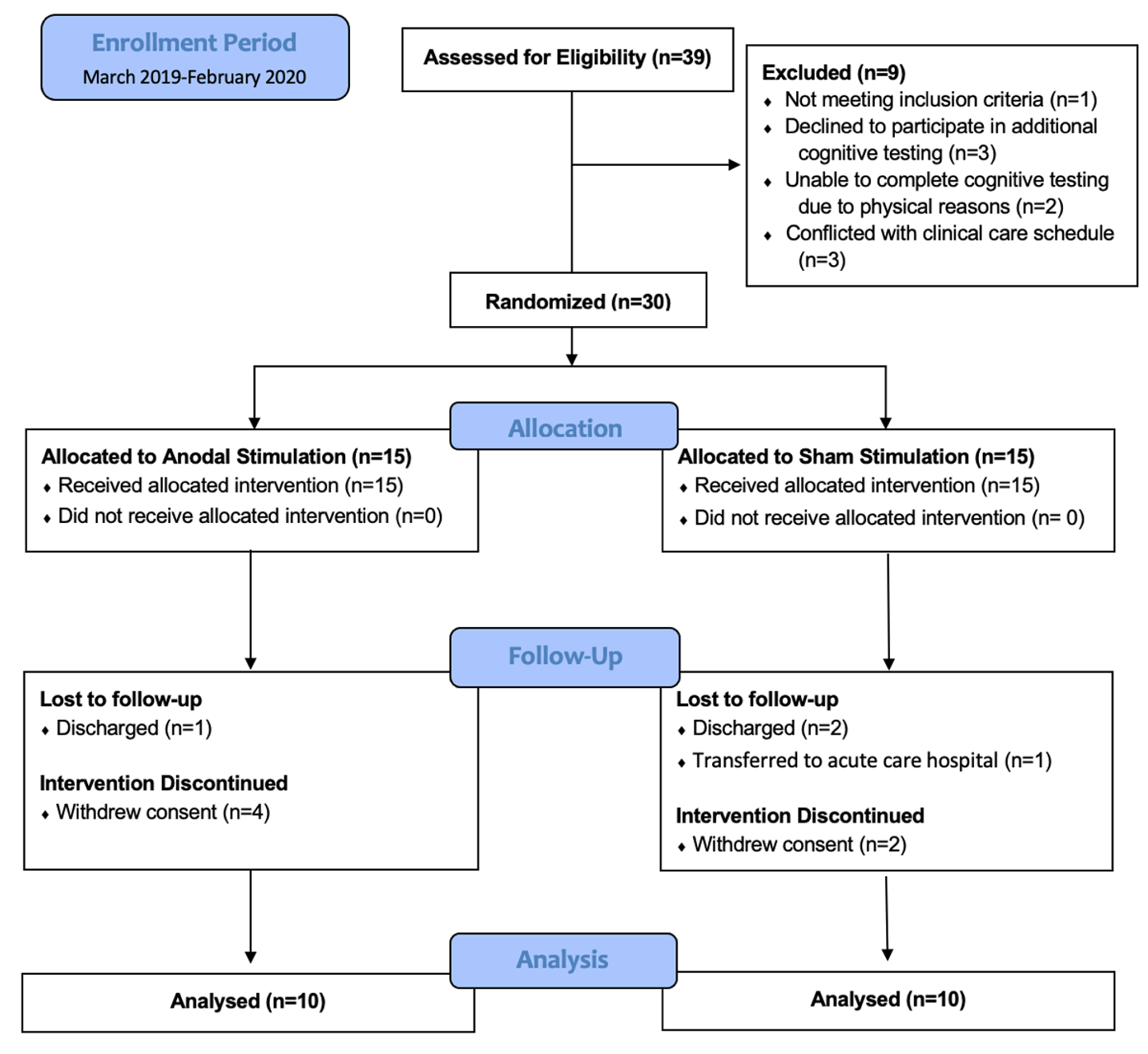
**

Adapted from the CONSORT 2010 Statement [79]

79. Schulz KF, Altman DG, Moher D. CONSORT 2010 statement: Updated guidelines for reporting parallel group randomized trials. *Ann Intern Med* (2010) 152:726–732. doi:10.7326/0003-4819-152-11-201006010-00232
